# Association of Ethnicity With Multisystem Inflammatory Syndrome in Children Related to SARS-CoV-2 Infection: An International Case-Referent Study

**DOI:** 10.3389/fped.2021.707650

**Published:** 2021-10-15

**Authors:** Judith G. Middelburg, Thomas E. M. Crijnen, Lorenzo D'Antiga, Lucio Verdoni, Ashish Chikermane, Padma Garg, Bhaswati C. Acharyya, Giulia Pruccoli, Aviad Schnapp, Abdul Rauf, Rutger A. Middelburg

**Affiliations:** ^1^Department of Medical Science, Middelburg Solutions, Oegstgeest, Netherlands; ^2^Paediatric Intensive Care Unit, Ziekenhuis Netwerk Antwerpen Queen Paola Children's Hospital, Antwerp, Belgium; ^3^Paediatric Department, Hospital Papa Giovanni XXIII, Bergamo, Italy; ^4^Department of Cardiology, Birmingham Women's and Children's NHS Foundation Trust, Birmingham, United Kingdom; ^5^Division of Pediatric Critical Care Medicine, University of Mississippi Medical Center, Jackson, MS, United States; ^6^Division of Pediatric Infectious Disease, University of Mississippi Medical Center, Jackson, MS, United States; ^7^Division of Pediatric Cardiology, University of Mississippi Medical Center, Jackson, MS, United States; ^8^Department of Pediatric Gastroenterology, AMRI Hospitals, Kolkata, India; ^9^Department of Pediatrics, AMRI Hospitals, Kolkata, India; ^10^Department of Pediatric Cardiology, AMRI Hospitals, Kolkata, India; ^11^Department of Pediatric and Public Health Sciences, Regina Margherita Children's Hospital, University of Turin, Turin, Italy; ^12^Department of Pediatrics, Hadassah Medical Center, The Faculty of Medicine, Hebrew University of Jerusalem, Jerusalem, Israel; ^13^Department of Pediatrics, Baby Memorial Hospital, Calicut, India; ^14^Department of Clinical Epidemiology, Leiden University Medical Center, Leiden, Netherlands

**Keywords:** COVID-19, SARS-CoV2, MIS-C, PIMS, ethnicity

## Abstract

**Background:** It has been suggested that children and infants can develop multisystem inflammatory syndrome in children (MIS-C) in response to a SARS-CoV-2 infection and that Black children are overrepresented among cases. The aim of the current study was to quantify the association between Black, Asian, or other non-White genetic background and COVID-19-related MIS-C in children and infants.

**Methods:** Eight different research groups contributed cases of MIS-C, potentially related to SARS-CoV-2 infection. Several sensitivity analyses were performed, including additional data available from the literature. Analyses were stratified by geographical region.

**Results:** Seventy-three cases from nine distinct geographical regions were included in the primary analyses. In comparison to White children, the relative risk for developing MIS-C after SARS-CoV-2 infection was 15 [95% confidence interval (CI): 7.1 to 32] for Black children, 11 (CI: 2.2 to 57) for Asian, and 1.6 (CI: 0.58 to 4.2) for other ethnic background.

**Conclusion:** Pediatricians should be aware of the fact that the risk of COVID-19-related MIS-C is severely increased in Black children.

## Introduction

Since the start of the outbreak of SARS-CoV-2 in December 2019, in the Hubei province in China, the virus has spread across the entire world (1–3). It was quickly reported that children were relatively unlikely to be severely affected by COVID-19, the disease caused by SARS-CoV-2 (4–7). Shortly after the worst peak in COVID-19 in adults, reports from Europe started to appear about a Kawasaki-like syndrome that occurred in children and infants and seemed to be linked to COVID-19 (8). In some of these case series, Black patients seemed to be overrepresented (9–13). However, none included a control group that allowed for quantification of this overrepresentation.

Due to the incomplete presentation of this Kawasaki-like syndrome in the presence of SARS-CoV-2 infection, it was soon renamed pediatric inflammatory multisystem syndrome (PIMS) or multisystem inflammatory syndrome in children (MIS-C).

Due to the similarity of MIS-C and (atypical) Kawasaki disease, it is interesting to note, that the incidence of Kawasaki disease in the absence of SARS-CoV-2 infection has been estimated to be two times higher in Black children than in White children (14, 15). This raises the possibility that an increased incidence of MIS-C, closely resembling Kawasaki disease, in the former group was also to be expected following SARS-CoV-2 infection. For Asian children, the incidence of Kawasaki disease in the absence of SARS-CoV-2 infection is estimated to be between two and 30 times higher, depending on the country or region of Asia considered (14, 16). However, for MIS-C following SARS-CoV-2 infection, overrepresentation of Asian children has not yet been reported.

If Black children are indeed affected disproportionately, this could also shed new light on the high incidence of severe COVID-19 reported in Black adults of (17–19). A systematic review suggested that not only Black adults, but all Black, Asian, and Minority Ethnic (BAME) individuals are at increased risk of infection and subsequently have worse clinical outcomes, compared to white individuals (20, 21). Currently, it remains unclear whether, and to what extent, different ethnic groups are overrepresented amongst children and infants, with MIS-C after SARS-CoV-2 infection.

The aim of the current study was therefore to compile and quantify the available evidence for an association between Black, Asian, or other non-White genetic background and COVID-19-related MIS-C in children and infants.

## Methods

### Data

#### Cases

Most included cases have previously been reported without a reference group (Table 1). All cases clinically judged to be MIS-C in any of the participating centers were included. Participating centers were: Hospital Papa Giovanni XXIII, Bergamo, Italy; Birmingham Women's and Children's NHS Foundation Trust, Birmingham, United Kingdom; University of Mississippi Medical Center, Jackson, USA; AMRI Hospitals, Kolkata, India; Regina Margherita Children's Hospital, Turin, Italy; Hadassah Medical Center, Jerusalem, Israel; Baby Memorial Hospital, Calicut, India. Ethnicity was categorized, by the reporting doctor, as being Black, Asian, White, or other. The category other included all categories reported in numbers insufficient to allow for separate analyses (e.g., South American, Middle Eastern, Native American, Hawaiian, and Romani).

**Table 1:** Numbers of reported cases by participating center.

**Region**	**Black**	**Asian**	**White**	**Other**	**Total**	**References**
Bergamo, Italy	4		17	1	22	Verdoni et al. (8)^a^
Paris, France	10		4	2	16	Pouletty et al. (10)
Birmingham, UK	5	3		1	9	Ramcharan et al. (9)
West Midlands, UK	3	3			6	Ramcharan et al. (9)
Mississippi, USA	7		1	1	9	Rivera-Figueroa et al. (22)^a^
Kolkata, India		5			5	Acharyya et al. (23)
Turin, Italy			4		4	Licciardi et al. (24)^a^
Calicut, India		1			1	Rauf et al. (25)
Jerusalem, Israel				1	1	Schnapp et al. (26)
Total (primary analyses)	29	12	26	6	73	
England, UK	22	18	12	6	58	Whittaker et al. (27)
New York state, USA	31	4	29	14	78	Dufort et al. (12)
26 states, USA	46		35	66	147	Feldstein et al. (13)^b^
Total (including literature data)	128	34	102	92	356	

a *Additional cases included after original publication*.

b *In the original publication the numbers presented for different categories (Table 1 of that publication) don't add up to the total reported in that publication. Therefore, the category specific numbers were used and the total was ignored during data-extraction*.

#### Reference

For each case or case series for which data on the ethnicity of cases could be retrieved, a reference value was also obtained. These reference values represent the expected percentage of Black, Asian, White, or other cases, according to the specific geographic regions where cases originated from. The observed percentages among cases were compared to these reference values. Reference values were either supplied by the author supplying the case information or estimated based on publicly available demographic data for the relevant region (see Supplemental Material for more details, also on the websites providing the data and the categories of ethnicity reported there).

## Statistical Analyses

All cases for which both data on ethnicity of cases and a reliable reference distribution could be obtained were included in the primary analyses. The analyses were corrected for the region of origin of the cases. To quantify the strength of the association of ethnicity and COVID-19-related MIS-C, Mantel-Haenszel weighted, pooled odds ratios with 95% confidence intervals were calculated, according to published formulas (28, 29). Due to the method of selecting the reference distribution (i.e., as an estimation of the distribution in the entire source population of cases), these odds ratios directly estimate relative risks, and are interpreted and presented as such throughout (30).

## Sensitivity Analyses

Three sensitivity analyses were performed. These analyses included data from the literature, to be better able to appreciate the external validity of our results. For this purpose, we selected the three largest case series reported at the time of our analyses (i.e., August 2020).

### Sensitivity Analyses 1

One large published case series, which reported ethnicity, included cases from eight hospitals across three different urbanized areas in England (27). This case series included cases across several distinct geographic regions, without a clear registration of which cases originated from which region. This made it impossible to obtain reliable reference values for this series.

To appreciate the effect of including a large number of cases with a poorly representative reference value, we performed two sets of sensitivity analyses including these data. Together, these two sets of sensitivity analyses provide the upper and lower bounds between which the relative risk would be expected to have been estimated, if it would have been possible to obtain a reliable reference value for this large case series.

First, the whole of England was taken as a reference. This will almost certainly provide an underestimation of the percentage of non-White people, compared to the urbanized setting where the cases arose from. Therefore, the relative risk for all non-White groups will be overestimated in these analyses.

### Sensitivity Analyses 2

Second, as the opposite extreme, the borough of Lewisham (London) was taken as a reference. This is the district with the highest percentage of non-White inhabitants in England. It will therefore, by definition, result in an overestimation of the percentage of non-White people. Therefore, the relative risks for the non-White groups will be underestimated in these analyses.

### Sensitivity Analyses 3

Two large American case series were published shortly after we completed our primary analyses, one from New York State and one from 26 states across the entire United Stated of America (USA) (12, 13). Since these were such large case series, we therefore performed a third set of sensitivity analyses, where the published data from these two case series were included in our analyses. For these sensitivity analyses we took the state of New York and the whole of the USA as the respective references for these two case series.

## Results

### Cases

Necessary information on ethnicity of both cases and a reference value could be obtained for a total 73 cases from nine distinct geographical regions. Numbers of included cases, according to their geographical region and their ethnicity, are show in Table 1.

With 29 cases (40%), Black children formed the biggest group amongst cases. White children formed the second biggest group with 26 cases (36%). In the reference populations Black children were always a minority, ranging from 0.4 to 38%. Even in the reference population with 38% Black, this was still a minority compared to 59% White.

### Ethnic Background and MIS-C

The association between ethnic background and MIS-C linked to SARS-CoV-2 infection, is shown in Table 2. The relative risk for developing MIS-C after SARS-CoV-2 infection, compared to White children, was 15 (95% confidence interval (CI): 7.1 to 32) for Black children, 11 (CI: 2.2 to 57) for Asian children, and 1.6 (CI: 0.58 to 4.2) for children with other non-White ethnic background.

**Table 2:** Relative risk of MIS-C by ethnicity.

	**Primary** ^**a**^			**Sensitivity 1** ^**b**^			**Sensitivity 2** ^**c**^			**Sensitivity 3** ^**d**^		
	***n***	**RR**	**95% CI**	**n**	**RR**	**95% CI**	***n***	**RR**	**95% CI**	***n***	**RR**	**95% CI**
White	26	1	reference	38	1	reference	38	1	reference	90	1	reference
Black	29	15	(7.1 to 32)	51	22	(13 to 38)	51	6.8	(4.2 to 11)	106	6.9	(5.1 to 9.3)
Asian	12	11	(2.2 to 57)	30	15	(7.7 to 30)	30	9.1	(4.7 to 18)	16	1.3	(0.64 to 2.7)
Other	6	1.6	(0.58 to 4.2)	12	3.2	(1.6 to 6.1)	12	2.0	(1.0 to 4.0)	86	13	(9.9 to 17)
Total	73			131			131			298		

a *The primary analyses, including only data from the participating centers*.

b *Sensitivity analyses 1, including data from Whittaker et al. (27) taking the whole of England as the reference value (resulting in overestimation of the relative risk)*.

c *Sensitivity analyses 2, including data from Whittaker et al. (27) taking the borough of Lewisham (London) as the reference value (resulting in underestimation of the relative risk)*.

d *Sensitivity analyses 3, including data from two large American case series (12, 13). n, number of included cases. RR, relative risk. 95% CI, 95% confidence interval*.

### Sensitivity Analyses and Supplementary Material

Results for the sensitivity analyses are shown in Table 2.

The Supplementary Material shows the reference values used for the different regions and how they were obtained.

## Discussion

This study shows that Black children are 15 times more likely than White children to develop COVID-19-related MIS-C. Asian children are 11 times more likely and other non-White children do not have a substantially elevated risk.

Especially the high risk among Black children is noteworthy, for two reasons. First, it is over seven times higher than any previous estimate of the association of normal Kawasaki disease (i.e., not associated with SARS-CoV-2 infection) and Black children (14, 15). Second, it is also five times as high as the overrepresentation of Black American and Latino adults which has been noted for both severe COVID-19 and COVID-19 related mortality (18–20). This overrepresentation among adults has generally been linked to pre-existing health disparities, which are known to affect both these populations. If the overrepresentation among children was due to the same health disparities alone, it would be expected to be of similar magnitude. Instead, it is much more pronounced, suggesting other factors are likely to contribute as well. These other factors could include further health disparities, environmental factors, and also a possible contribution from a genetic predisposition to the occurrence of a hyperinflammatory response.

Such a genetic component could be more obvious in children, where the baseline risk of a severe course of COVID-19 is much lower than in adults (4, 5, 7). A genetic predisposition among Black children could also explain in part why the Kawasaki-like syndrome has not been reported in China, where Black people are much less common (4, 5). Future studies should aim to further elucidate whether Black children indeed have a genetic predisposition toward, an hyperinflammatory response and how specific such a response is for COVID-19.

This study has three main limitations. The first limitation is that we included data from all cases of MIS-C possibly linked to SARS-CoV-2 infection, based on clinical evaluation, without any specific requirements for laboratory test results. We therefore possibly also included some cases which were entirely unrelated to SARS-CoV-2 infection. Such a misclassification of cases could result in an overestimation of the association for Asian children, since Asian ethnicity is known to be associated very strongly with common Kawasaki disease. Therefore, the observed association in this group could, at least in part, be due to Kawasaki disease unrelated to SARS-CoV-2 infection. However, for Black children, who have only a double risk of common Kawasaki disease, this misclassification is likely to lead to an underestimation of the true association. The fact that we still observed a very strong association therefore suggests that either this misclassification problem was very small, or the true association was even stronger than observed in this study.

The second limitation is that the reference groups relied mostly on census data. Although we carefully selected the most appropriate census for each region, and we split cases from one center into two distinct regions of origin, there is still a chance that these data were not entirely representative of the source population of the cases. This could lead to some inaccuracy in our estimates. However, we did find reasonably reliable reference values for most regions. Furthermore, we performed sensitivity analyses, including pertinently inaccurate reference values for the large case series from England, which we included from the literature (27). These sensitivity analyses result in slightly different effect estimates, but not in wildly different overall conclusions.

The third limitation is that we did not include South American, or Hispanic backgrounds as a separate group in our analyses. This group, Arabs, and other ethnic minority groups are all lumped together in the category of “other.” Although it might have been interesting to investigate potential differences between these subgroups of “other,” the available data did not allow for this distinction to be made reliably. We therefore opted for pooling these groups together instead. Since, in our primary analyses, we saw no substantial increase in risk for this group, it seems likely that none of the ethnic groups pooled together in these analyses have risks as high as the Black children. However, we cannot rule out entirely that a relevant association for a minor group is obscured by pooling that group with a larger group without an association. Furthermore, sensitivity analyses 3 shows markedly different results for this group specifically, with a 13 times increased risk. This sensitivity analyses derives most of its data from two large American case series. These two series include many Hispanic and Latino cases, which are almost completely absent from the “other” category in all other analyses. It therefore seems likely that this group is also at a greatly increased risk. However, based on our data, no reliable conclusions on this subject are possible. Further research into this association, however, seems warranted.

While the report of the current study was under review for publication, another study was published, which investigated the same research question (31). This study observed a higher incidence of MIS-C among Black and Hispanic children in New York City. Due to a different study design, a direct comparison of the results is difficult to make. However, the results seem to confirm at least our observation of the presence of an association in both Black and Hispanic children (31).

Two additional potential limitations could be the number of cases and the availability of further clinical and demographic data. In epidemiological studies, such as this one, it is always desirable to have more data. With thousands of MIS-C cases diagnosed world-wide, 73 might seem like a small number. However, as reflected by the presented confidence intervals, these 73 cases were more than sufficient to estimate the association with ethnicity. Further, ethnicity is often not recorded as part of routine clinical care. This makes it difficult to include many of the diagnosed case, without the introduction of selection bias, if ethnicity was reported selectively. To avoid bias, we have therefore chosen to limit our data collection to hospitals that could report all the necessary data for all their cases. We initially focused on the data presented in this report. After completion of the study, additional clinical and demographic data could no longer be retrieved for all included cases. This limits the ability to fully appreciate the representativeness of these cases for MIS-C cases in general. However, we included all clinically diagnosed cases from the participating centers, without selection. Therefore, these cases are likely to be broadly representative of clinically diagnosed MIS-C during the COVID-19 pandemic.

## Conclusions

Black children have a greatly increased risk of COVID-19-related MIS-C, which appears to occur shortly after the peak in adult COVID-19. Pediatricians, especially those working in hospitals with large Black populations should be extra vigilant in the aftermath of the COVID-19 epidemic among adults. A similar, though weaker, association was observed for Asian children. However, this association could be partly due to the association with common Kawasaki disease, which is well known to be present in this group. Hispanic and Latino children could be at increased risk as well. However, the data included in this study is inconclusive on this subject.

## Data Availability Statement

The original contributions presented in the study are included in the article/Supplementary Material, further inquiries can be directed to the corresponding authors.

## Ethics Statement

The studies involving human participants were reviewed and approved by Institutional Review Board of the LUMC for observational studies. Written informed consent from the participants legal guardian/next of kin was not required to participate in this study in accordance with the national legislation and the institutional requirements.

## Author Contributions

JM conceptualized and executed the research, drafted the initial manuscript, and reviewed the revised manuscript. TC conceptualized the research, critically revised the manuscript, and reviewed the revised manuscript. LD'A, LV, AC, PG, BA, GP, AS, and AR provided data, critically revised the manuscript, and reviewed the revised manuscript. RM conceptualized, designed, and executed the research, critically revised the manuscript, reviewed the revised manuscript, affirms that the manuscript is an honest, accurate, transparent account of the study being reported, that no important aspects of the study have been omitted, and that any discrepancies from the study as originally planned have been explained. All authors approved the final manuscript as submitted and agree to be accountable for all aspects of the work.

## Conflict of Interest

The authors declare that the research was conducted in the absence of any commercial or financial relationships that could be construed as a potential conflict of interest.

## Publisher's Note

All claims expressed in this article are solely those of the authors and do not necessarily represent those of their affiliated organizations, or those of the publisher, the editors and the reviewers. Any product that may be evaluated in this article, or claim that may be made by its manufacturer, is not guaranteed or endorsed by the publisher.
