# Supplementary material for: Association of Ethnicity With Multisystem Inflammatory Syndrome in Children Related to SARS-CoV-2 Infection: An International Case-Referent Study
Source: Front Pediatr. 2021 Oct 15;9:707650. doi: 10.3389/fped.2021.707650 (PMC8555018; doi:10.3389/fped.2021.707650)
Supplement: Supplementary file 1 [file Data_Sheet_1.PDF]

Table S1

Region	Black	Asian	White	Other	Total	Source
Bergamo, Italy[a]	15585	19431	1011485	68089	1114590	https://ugeo.urbistat.com/AdminStat/en/it/demografia/stranieri/bergamo/16/3
Paris, France	4101	201	14921	7276	26499	https://parisedc.com/population-by-raceethnicity
Birmingham, UK[b]	231400	691600	1505400	171600	2600000	Office of National Statistics: https://www.ons.gov.uk
West Midlands, UK[b]	194700	637200	4784900	194700	5811500	Office of National Statistics: https://www.ons.gov.uk
Mississippi, USA	1124984	32738	1758904	59523	2976149	United States Census Bureau: https://www.census.gov/quickfacts/fact/table/MS/PST045219
Kolkata, India[c]		1000				Author's estimate of normal admission numbers
Turin, Italy[a]	9556	16878	2051178	181911	2259523	https://ugeo.urbistat.com/AdminStat/en/it/demografia/stranieri/torino/1/3
Jerusalem, Israel[d]	32	18	111	838	1000	https://www.indexmundi.com/israel/ethnic_groups.html
Calicut, India[b]	5	990	5		1000	Author's estimate of normal admission numbers
England, UK[e]	1864890	4213531	48209395	1788096	56075912	https://www.ethnicity-facts-figures.service.gov.uk
[f]	81600	27900	160800	30000	300300	https://en.wikipedia.org/wiki/London_Borough_of_Lewisham
26 states, USA[g]	43984096	19366132	250446756	14114299	327911283	https://www.census.gov/quickfacts/fact/table/US/PST045219
New York state, USA[g]	3423826	1750820	13539678	739235	19453561	https://www.census.gov/quickfacts/fact/table/NY/PST045219

Numbers represent numbers of individuals in the reference population.

a. Reclassified into ethnic groups, from the countries of origin reported that were on the website (immigrants from North African countries were classified as "Other", to be more in line with the UK data, which distinguished "Black" and "Arab" as separate categories).

b. Categories "Mixed Race", "Arab", and "Other Ethnic Heritage" were re-classified into "Other".

c. Only percentages were provided; therefore the total population size was arbitrarily set to 1,000.

d. Percentages on the website were recalculated according to the author's estimate of 30% non-Jewish population in the hospital. Only percentages were provided; therefore the total population size was arbitrarily set to 1,000.

e. England and Wales: Sensitivity analyses 1.

f. London, borough of Lewisham: Sensitivity analyses 2.

g. Included only in sensitivity analyses 3.
